# Convolutional Neural Network with Attention Mechanism and Visual Vibration Signal Analysis for Bearing Fault Diagnosis

**DOI:** 10.3390/s24061831

**Published:** 2024-03-13

**Authors:** Qing Zhang, Xiaohan Wei, Ye Wang, Chenggang Hou

**Affiliations:** 1 School of Instrument Science and Technology, Xi’an Jiaotong University, Xi’an 710049, China; zhangq@mail.xjtu.edu.cn; 2 School of Mechanical Engineering, Xi’an Jiaotong University, Xi’an 710049, China; weixiaohan123@stu.xjtu.edu.cn (X.W.); aii@mail.xjtu.edu.cn (Y.W.)

**Keywords:** attention mechanism, convolutional neural networks, bearing, fault diagnosis

## Abstract

Bearings, as widely employed supporting components, frequently work in challenging working conditions, leading to diverse fault types. Traditional methods for diagnosing bearing faults primarily center on time–frequency analysis, but this often requires expert experience for accurate fault identification. Conversely, intelligent fault recognition and classification methods frequently lack interpretability. To address this challenge, this paper introduces a convolutional neural network with an attention mechanism method, denoted as CBAM-CNN, for bearing fault diagnosis. This approach incorporates an attention mechanism, creating a Convolutional Block Attention Module (CBAM), to enhance the fault feature extraction capability of the network in the time–frequency domain. In addition, the proposed method integrates a weight visualization module known as the Gradient-Weighted Class Activation Map (Grad-CAM), enhancing the interpretability of the convolutional neural network by generating visual heatmaps on fault time–frequency graphs. The experimental results demonstrate that utilizing the dataset employed in this study, the CBAM-CNN achieves an accuracy of 99.81%, outperforming the Base-CNN with enhanced convergence speed. Furthermore, the analysis of attention weights reveals that this method exhibits distinct focus of attention under various fault types and degrees. The interpretability experiments indicate that the CBAM module balances the weight allocation, emphasizing signal frequency distribution rather than amplitude distribution. Consequently, this mitigates the impact of the signal amplitude on the diagnostic model to some extent.

## 1. Introduction

Serving as essential support components for rotating parts, rolling bearings find widespread application in various fields, including industrial manufacturing, wind power, rail transit, and aerospace [1,2,3,4]. However, the prevalence of uninterrupted loading in harsh working conditions substantially elevates the risk of bearing failures [5,6,7]. Traditional approaches to bearing fault diagnosis typically entail the analysis of bearing signals in the time and frequency domains, often relying on manual techniques to discern fault features [8,9,10,11,12,13,14,15,16]. Nevertheless, bearings exhibiting faults at different locations, and varying severities manifest distinct signal characteristics that are indicative of the specific fault [17,18,19]. Conventional fault diagnosis methods encounter challenges in accurately extracting fault features due to the intricate nature of these differences. Consequently, the establishment of an efficient intelligent bearing fault diagnosis method becomes imperative.

In response to the advancements in artificial intelligence technology and the proliferation of extensive monitoring data, numerous data-driven methodologies for bearing fault diagnosis have emerged. In recent years, there has been a notable surge in the adoption of deep neural networks, particularly leveraging their capacity to circumvent manual feature extraction and selection processes through an end-to-end approach, within the domain of bearing fault diagnosis. Among the various deep learning algorithms, convolutional neural networks (CNNs) have gained widespread acceptance and application in fault diagnosis within the field due to their capacity to address data in different dimensions. Zhang et al. introduced a one-dimensional CNN designed specifically for the diagnosis of bearing vibration signals [20]. This approach aims to capture the fault characteristics of the bearing throughout continuous rotation periods. Li et al. employed a convolutional neural network for the diagnosis of the two-dimensional time–frequency graph of bearing fault signals. Additionally, InceptionResNetV2 was employed to accommodate the non-rigid characteristics and larger receptive field in the graph [21]. Łuczak proposed a fault diagnosis method using a CNN to process spectrograms for fault diagnosis [22]. In this method, short-time Fourier transform (STFT) is applied to transform 6 DOF sensor data into spectrograms that are combined into an RGB image. He also proposed a method which extends the application of CNNs to the recognition of specially crafted time–frequency images [23]. This method extracts features from the original signal by CWT and subsequently utilizes a CNN for fault diagnosis. Kim et al. introduced an end-to-end convolutional model for fault diagnosis with three-axis vibration signals [24]. This model uses a grouped convolution to extract the feature maps of each axis. Nonetheless, when the signal is influenced by noise stemming from operational conditions, the distinctions among bearing fault signal samples may be obscured by variations in features. This poses a challenge for convolutional neural networks (CNNs) to effectively differentiate. Therefore, enhancing the discriminative capabilities of CNNs in distinguishing bearing faults becomes a critical consideration.

Drawing inspiration from the principles governing human attention, the attention mechanism has been introduced into the realm of deep learning. This mechanism directs machine learning methods to concentrate on crucial data information through weight distribution, effectively disregarding less pertinent information. The attention mechanism has gradually become the state of the art and has affected the direction of the field of bearing fault diagnosis. Wang et al. introduced a method for bearing fault diagnosis, utilizing a convolutional neural network integrated with a channel attention mechanism to analyze vibration signal image samples that were generated through the symmetrized dot pattern (SDP) method [25]. The experimental findings demonstrated that the incorporation of the attention mechanism notably augmented the CNN’s efficacy in diagnosing image samples. Li et al. proposed a deep learning-based bearing fault diagnosis method that integrates an attention mechanism. In this approach, a convolutional neural network is employed to extract features from the original bearing vibration signal sequence. Subsequently, a single-layer network structure is utilized in the feature classification stage to generate attention weights [26]. The experimental results indicated that the diagnosis method incorporating the attention mechanism exhibited superior accuracy and efficiency compared to the method that was lacking attention. Zhao et al. presented an enhanced method for CNN-based bearing fault diagnosis [27]. This method incorporated a multi-scale convolution kernel for feature extraction and introduced a channel attention mechanism. The experimental results demonstrated that the attention mechanism effectively enhanced the relevant fault information adaptively, while suppressing interference information.

While deep learning technology has exhibited commendable performance in bearing fault diagnosis, it is predominantly considered a black-box model within the fault diagnosis domain. The inherent challenge lies in explaining the relationship between the extracted features’ meaning as assigned by the model and the underlying fault mechanism. Consequently, there is a pressing need for research on the interpretability of fault diagnosis methods employing deep learning techniques. Yang et al. addressed this issue by applying neuron activation maximization and saliency map methods to the CNN-based bearing fault diagnosis task [28]. Their methodology revealed that low-level spatial features, such as signal peaks, are extracted by the initial convolutional layers. As the network’s layers progress, the fully connected layer extracts higher-level and more intricate abstract features that encapsulate sequential information, which is subsequently utilized for effective fault diagnosis. This approach contributes to a deeper understanding of the interpretability aspects of deep learning in the context of bearing fault diagnosis.

In previous research, it was observed that diagnostic methods incorporating attention mechanisms are primarily applied to one-dimensional data channels. The comprehensive application of attention mechanisms to multi-dimensional data is largely overlooked. Additionally, a challenge exists in interpreting the correlation between existing methods and their corresponding diagnosis results. To augment the accuracy of bearing fault diagnosis and the interpretability of the CNN, this paper proposed a CNN with an attention mechanism and a visual vibration signal analysis. The key contributions of this work are summarized as follows:

(1) This paper introduces a pioneering CNN-based bearing fault diagnosis method that incorporates an attention mechanism, utilizing time–frequency diagrams as input. The method achieves precise fault diagnosis through an end-to-end approach, eliminating the need for time-consuming manual feature extraction and professional expertise.
(2) The proposed method integrates an attention mechanism termed the Convolutional Block Attention Module (CBAM). This module enhances the network’s capability for fault feature extraction and fortifies its robustness across diverse operating conditions.
(3) Additionally, the proposed method incorporates a weight visualization module known as the Gradient-Weighted Class Activation Map (Grad-CAM). This module enhances the interpretability of the convolutional neural network by generating a visualized heat map of the fault’s time–frequency diagram, providing insights into the regions that are crucial for fault diagnosis.

The rest of this article is organized as follows: Section 2 briefly introduces the theory of the CBAM and the Grad-CAM. Section 3 describes the proposed method in detail. Section 4 evaluates the method’s performance on the simulated bearing failure dataset. Section 5 concludes this article.

## 2. CBAM and Grad-CAM

### 2.1. CBAM

CBAM is a lightweight general module that can be seamlessly integrated into a CNN with negligible overhead and can be jointly trained by the end-to-end mode. After adding the attention weight calculation structure module, the weights are automatically generated during the training process of the deep learning method. Subsequently, the module assigns attention weights to the original data and affects the computational decision-making process of the deep learning method by changing the distribution of the original data. In this process, the learning ability of deep learning methods is improved.

In a CNN, distinct convolution kernels correspond to different channel outputs. Simultaneously, the features that are extracted by the network, as represented by these outputs, vary, and the significance of different features within the feature map group is not uniform. To address this, a channel attention module is incorporated into the convolutional network to emphasize various features within the feature map group.

**Figure 1:**
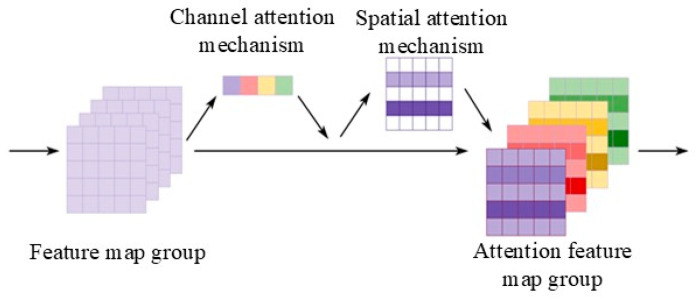
CBAM attention mechanism generation process.

Additionally, considering that the feature map itself is a two-dimensional image, a spatial attention module is introduced into the convolutional network to determine which parts of the feature image warrant focus. The process of attention generation is illustrated in Figure 1.

### 2.2. Grad-CAM

In the process of original data-to-result mapping, which is realized by the way of internal weight changes in machine learning, the learning process is affected by the attention information that is contained in the data directly. In order to explore the impact of the attention mechanism on the model, the Gradient-Weighted Class Activation Map (Grad-CAM), a weight visualization method for deep learning models, is introduced in the fault diagnosis method. As an advanced method for visualizing the weights of deep learning models, Grad-CAM can output a heat map of weights related to categories, which can clearly indicate the importance of the model’s weights to the sample by using different colors.

**Figure 2:**
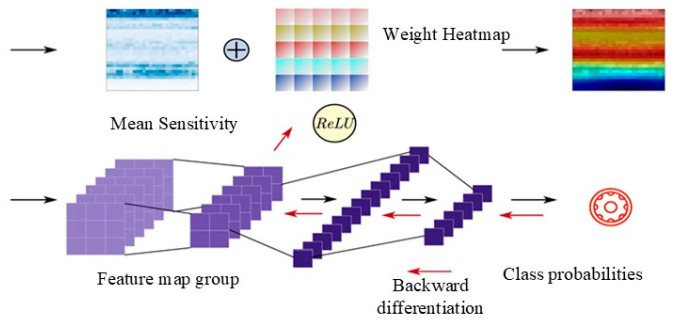
Grad-CAM heat map generation process.

Once the convolutional neural network (CNN) is trained, its capability to diagnose bearing fault signals is encapsulated in the form of internal weights, which are distributed across the convolutional network module and the fully connected classifier module. In order to comprehensively analyze the influence of internal weights, fault samples are overlaid onto the original two-dimensional time–frequency map. This overlaying process allows the generated weight heat maps to intuitively illustrate the attention of the fault diagnosis method to the fault information that is present in the time–frequency map. The detailed procedure of heat map generation is delineated in Figure 2.

## 3. Proposed Method

### 3.1. Architecture of Proposed Method

In this article, to improve the fault diagnosis accuracy as well as enhance the interpretability, an intelligent fault diagnosis method based on a convolutional neural network with an attention mechanism and visual analysis is proposed.

**Figure 3:**
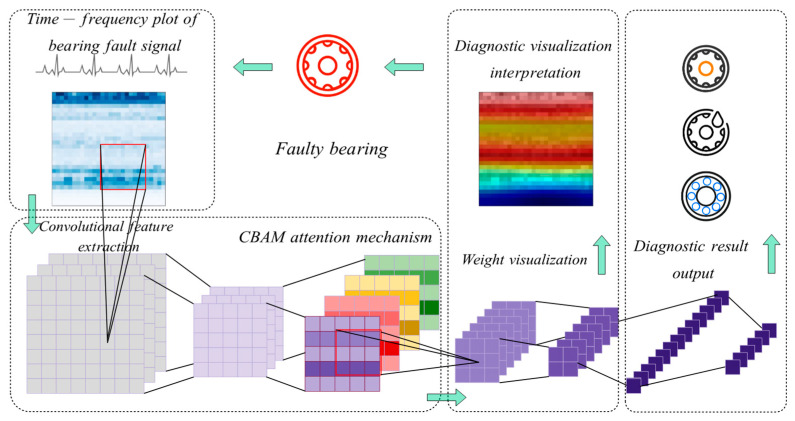
The proposed method based on CNN with attention mechanism.

Figure 3 shows the architecture of the proposed method, which mainly contains five components: a data preprocessing and input module, a convolution module, an attention module, a classifier, and a visualization module. First, in the data preprocessing and input module, the raw one-dimensional signal samples are transformed and compressed by the short-time Fourier transform. The two-dimensional time–frequency diagrams and the labels are divided into datasets. The second component is the convolution module, which performs the feature extraction automatically. The third component is the attention module, including the channel attention and the spatial attention, which calculate the attention weights automatically. Then, in the classifier component, the fault diagnosis results are obtained by using softmax in the fully connected layer. Finally, after the network has been trained, the attention heat map of the time–frequency diagram is generated from the visualization module. The structural parameters and training parameters of the proposed method are shown in Table 1 and Table 2, respectively.

### 3.2. Time–Frequency Graph Conversion

**Figure 4:**
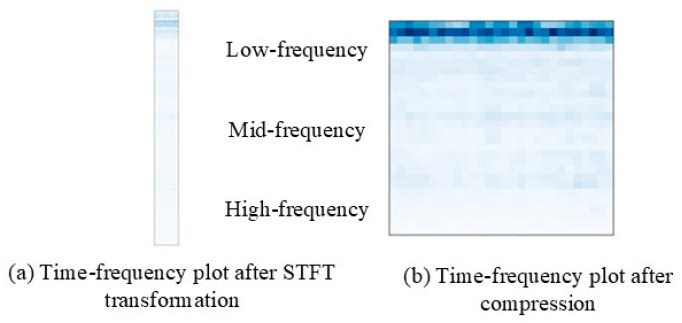
The time–frequency graph samples before and after processing.

To visualize the frequency band composition and distribution of bearing signals at various fault locations and severities, and to facilitate the exploration of the attention mechanism module’s role, along with the outcomes of the visual weight analysis, the proposed method employs a short-time Fourier transform (STFT) time–frequency diagram module. This module can transform the one-dimensional vibration signal into a two-dimensional time–frequency graph. The process of short-time Fourier transform is formulated as follows:

(1) $$STFT(t,f)=\int_{-\infty }^{\infty }x(\tau )h(\tau -t)e^{-j2\pi f\tau }d\tau$$

where x(τ) is the time domain signal, and h(τ−t) represents the window function. During the transformation, the length of the window affects the time and frequency resolution. As the window length increases, resulting in a longer intercepted signal, a trade-off emerges between the time resolution and the frequency resolution. Specifically, longer windows lead to a lower time resolution but a higher frequency resolution. In this paper, the Hanning window is chosen as the STFT window function, with a window width of 2048 points, and the overlap between the two windows is 50%. This choice ensures that each frame encompasses at least one complete rotation signal of the motor. To compress the original time–frequency map, an adaptive pooling module is employed, generating a time–frequency map with dimensions of 28 in length and width. Figure 4 illustrates the time–frequency graph samples before and after processing.

### 3.3. Model Training Process

**Figure 5:**
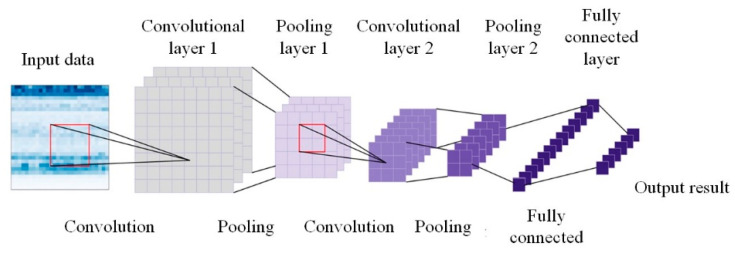
Feature map extraction process from the time–frequency graph.

For the sample input of the time–frequency graph, the convolutional neural network is used to extract the fault information from the time–frequency graph and generate the corresponding feature map group. The extraction process is shown in Figure 5.

For a given input feature map group F, the dimensional attributes are as follows: height H, width W, and number of channels C. The input form of the feature map group is denoted as $F\in R^{C\times H\times W}$. For the input feature map group F, average pooling and maximum pooling are performed, respectively, to realize the aggregation of information in the spatial dimension of the feature map group and generate two pooled feature maps, $F_{avg}^{c}$ and $F_{max}^{c}$.

**Figure 6:**
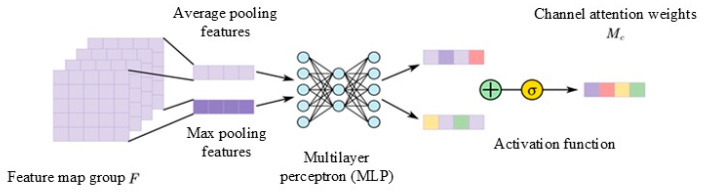
Channel attention generation process.

The two feature maps are subsequently input into a multilayer perceptron, each comprising a hidden neuron layer. This process results in the generation of two 1×1×C channel attention maps. In order to obtain a better nonlinear learning ability and reduce the number of parameters and computation, the number of neurons in the hidden layer is defined as C/r, in which r is the compression ratio. The corresponding elements of the two channel attention maps that are obtained by the multilayer perceptron are combined, and subsequently, the sigmoid activation function is applied to obtain the channel attention weights. The calculation process of the channel attention is shown in Figure 6. The attention weight generation process is shown in the following formula:

(2) $$M_{c}(F)=\sigma (MLP(AvgPool(F))+MLP(MaxPool(F)))$$

(3) $$M_{c}(F)=\sigma (W_{1}(W_{0}(F_{avg}^{c}))+W_{1}(W_{0}(F_{max}^{c})))$$

**Figure 7:**
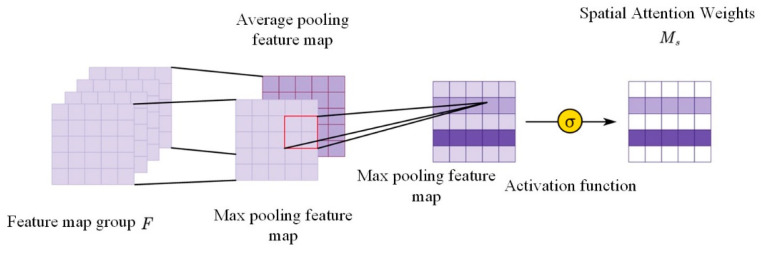
Spatial attention generation process.

In the channel direction of the feature map group, the information is aggregated through two pooling methods, and the maximum and average pooled feature maps $F_{avg}^{s}$ and $F_{max}^{s}$ are obtained; the dimension can be defined as 1×H×W. The two feature maps are spliced, and then, the convolution kernel with the 7×7 convolution area is used to perform the convolution operation on the feature map. Finally, the sigmoid activation function is used to obtain the spatial attention weights. The calculation process of the spatial attention is shown in Figure 7. The attention weights are calculated as follows:

(4) $$M_{s}(F)=\sigma (f^{7\times 7}([AvgPool(F);MaxPool(F)]))$$

(5) $$M_{s}(F)=\sigma (f^{7\times 7}([F_{avg}^{s};F_{max}^{s}]))$$

The entire attention calculation process is shown in the following formula:

(6) $$\begin{array}{l} F^{\prime }=M_{c}(F)⊗F,\\ F^{\prime \prime }=M_{s}(F^{\prime })⊗F^{\prime } \end{array}$$

where ⊗ is the Kronecker product operator.

### 3.4. Heat Map Generation

For a classified sample, the class probability output is denoted as $y^{c}$, and the final generated visualization result is denoted as $L_{Grad-CAM}^{c}$. In the first, partial derivatives for all channel feature maps, the output $F_{ij}^{k}$ is obtained by the last convolutional layer of the CNN model. Then, global average pooling on the feature maps is performed to generate the average sensitivity of the sample category to the feature map in the channel k’s output by the last layer of the convolutional layer. The calculation process is as follows:

(7) $$\alpha_{k}^{c}=\frac{1}{Z}\sum_{i}\sum_{j}\frac{\partial y^{c}}{\partial F_{ij}^{k}}$$

**Figure 8:**
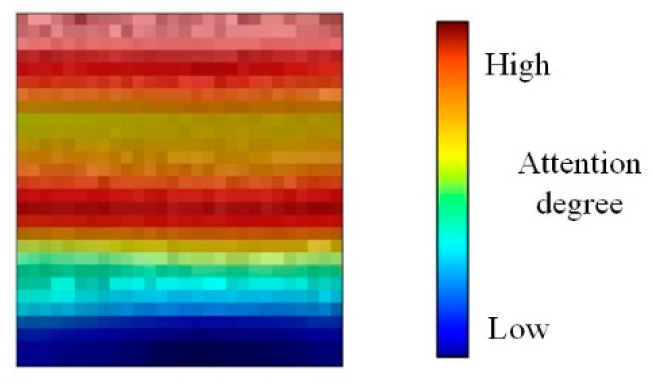
Heat map sample.

The partial derivative result represents the changing rate of the output concerning the input, indicating a unit change on the feature map. The output signifies how many units of change occurred and reflects the sensitivity of the output to the feature map. A higher gradient implies a greater sensitivity, suggesting that the pixel position is more likely to be associated with its category according to the model’s determination. Upon completing the calculation, it undergoes linear weighting with the corresponding channel to the feature map. Subsequently, the two-dimensional weighted heat map is generated through the activation function. Figure 8 illustrates a sample of the heat map, and the calculation process is as follows:

(8) $$L_{Grad-CAM}^{c}=ReLU(\sum_{k}\alpha_{k}^{c}F^{k})$$

## 4. Experimental Analysis

### 4.1. Dataset Generation

**Figure 9:**
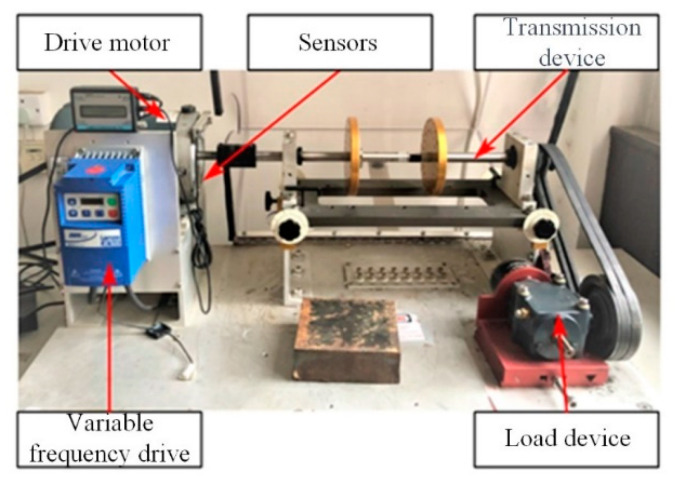
Bearing simulation experiment platform.

This experiment utilizes the MFS-Magnum motor fault comprehensive simulation experimental platform, manufactured by the company SQI, to simulate bearing faults. The experimental bearing platform designed for these simulation experiments is depicted in Figure 9. Three acceleration sensors, PCB PIEZOTRONICS 352C68, were installed in the horizontal and vertical radial directions of the motor front cover and the vertical radial direction of the motor, respectively, to collect the vibration signals. Three analog-to-digital conversion channels were set up for the synchronous acquisition of acceleration sensor signals. The sampling frequency was set at 12,800 Hz, and the sampling length was set at 15 s. Simulated fatigue spalling occurs due to the combined load bearing and relative rolling of both the inner and outer raceways, as well as the surfaces of the rolling elements. Under the influence of alternating loads, cracks initially form at locations experiencing maximum shear stress on the surface. Subsequently, these cracks expand to the contact surface, leading to the formation of spalling pits on the surface layer. Over time, this process progresses into the development of large flakes.

**Figure 10:**
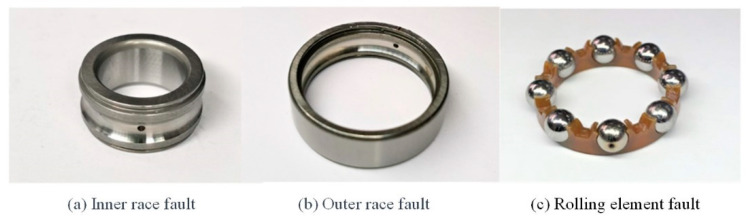
Three types of bearing fault manifestations.

In the experiment, three typical bearing faults located in the inner race, outer ring, and rolling element, respectively, which are shown in Figure 10, were simulated. Each type of fault includes four degrees of fault, controlled by the diameter of the sintering area of the laser at the fault point during processing, which are 0.8 mm, 1.0 mm, 1.2 mm, and 1.4 mm, respectively. The experiments in every fault type are executed with four different rotation frequencies, which are 10 Hz, 20 Hz, 30 Hz, and 35 Hz, and six different load levels. In this way, a total of 288 simulated operating states are generated. Five repetitions of each experiment are conducted under each fault state with the applied rotation frequency and load level. That is to say, the total number of samples in the dataset is 1440.

Due to the long collection time of a single fault signal, sliding window truncation was performed on the original signal to expand the number of samples in the dataset. We set the sliding window to a width of 8 s, which means that the window width was 102,400 points. To ensure coverage of all data and avoid the signal being too similar after truncation, the window sliding frequency was set to 18 times, and the step size was set to 5120 points. In total, 25,920 truncated data points were obtained, and the training set and validation set were split with a ratio of 80% and 20%. Finally, 20,736 truncated data points were obtained to comprise the training set, and 5184 truncated data points were obtained to form the validation set.

In the validation dataset, No. 1–1728 represent inner race fault data, No. 1729–3456 represent outer race fault data, and No. 3457–5148 represent rolling element fault data.

### 4.2. Attention Image Result and Performance Analysis

#### 4.2.1. Analysis of Bearing Fault Diagnosis Results

**Figure 11:**
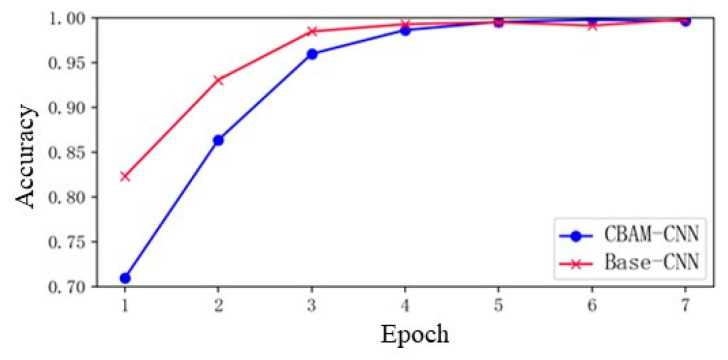
Accuracy curve of the CBAM-CNN and Base-CNN.

The performance of the bearing fault diagnosis method is analyzed in this section. The accuracy curve, depicting the proportion of correctly diagnosed samples among all samples for the CBAM-CNN with the CBAM module, is illustrated by the blue line in Figure 11. It can be seen that the CBAM-CNN achieves a diagnosis accuracy of nearly 100% on the bearing fault signal dataset, indicating that the proposed method has an excellent diagnostic ability for the bearing fault signal under the influence of multiple parameters. In order to verify the role of the attention module in the deep convolutional network, the Base-CNN without the CBAM module is trained under the same parameter conditions as a contrast. The diagnostic accuracy curve of the comparison model is shown in the red line in the figure.

The accuracy of the Base-CNN curve increases slightly faster than that of the CBAM-CNN curve, because the Base-CNN training process does not contain the calculation process of the CBAM module. However, it is worth noting that the CBAM-CNN achieved a rate of 0.9867 after four rounds of training, which is close to the accuracy of the Base-CNN, and reached a rate of 0.9981 after the sixth round of training, surpassing the Base-CNN’s accuracy rate.

**Figure 12:**
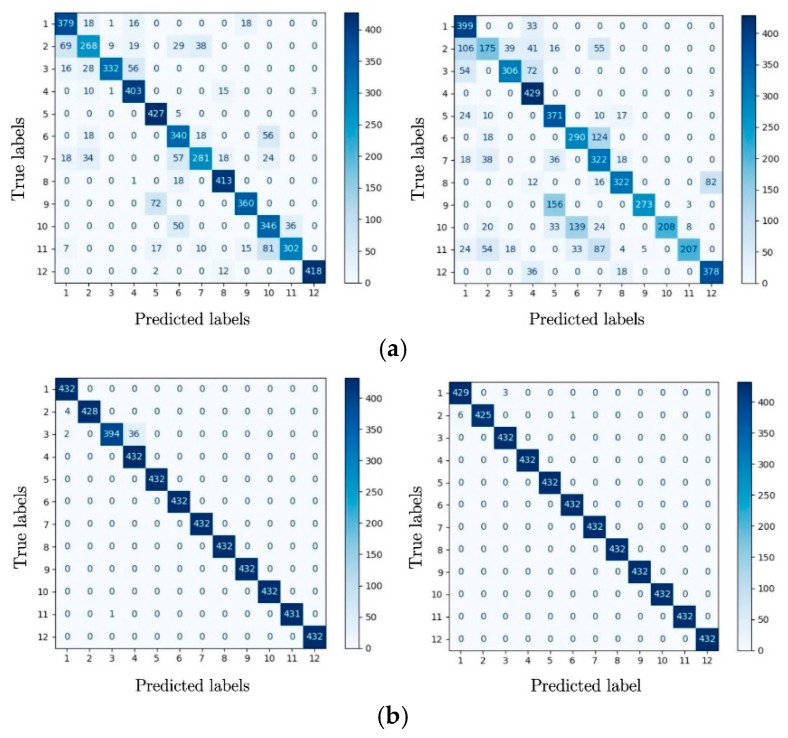
Confusion matrix formed in the first round and the sixth round. (**a**) depicts the confusion matrix for the first round, whereas (**b**) is for the sixth round. Each image presents the confusion matrix results for the Base-CNN on the left side and the CBAM-CNN on the right side. The true labels are from the validation set, while the predicted labels are the outputs of the CBAM-CNN and Base-CNN. Twelve labels are the combination of 3 types of bearing faults, which are inner race faults, outer race faults, and rolling element faults, and 4 rotation frequencies, which are 10 Hz, 20 Hz, 30 Hz, and 35 Hz. The values corresponding to the same true and predicted label indicate the number of correct predictions for that particular fault state category.

In order to analyze the classification performance of the model on each type of sample, the classification results of the two models in the first round and the sixth round are converted into the confusion matrix form, and the results are shown in Figure 12.

It can be seen that after the first training round, there are more classification errors in the classification results of the CBAM-CNN than those of the Base-CNN, because the independent computing structure of the CBAM module has not yet completed the learning of the differences between the fault signals. After the sixth round of training, the CBAM-CNN has only 10 sample classification errors, which is lower than the 42 of the Base-CNN. The CBAM-CNN achieves and exceeds the performance of the Base-CNN after only six rounds of training when additional attention weight calculation is required.

Therefore, the CBAM attention mechanism can improve the learning efficiency of a CNN. In addition, it can still achieve further performance improvement on the basis of the good performance of the CNN.

#### 4.2.2. Analysis of Bearing Fault Signal and Attention Weight

**Figure 13:**
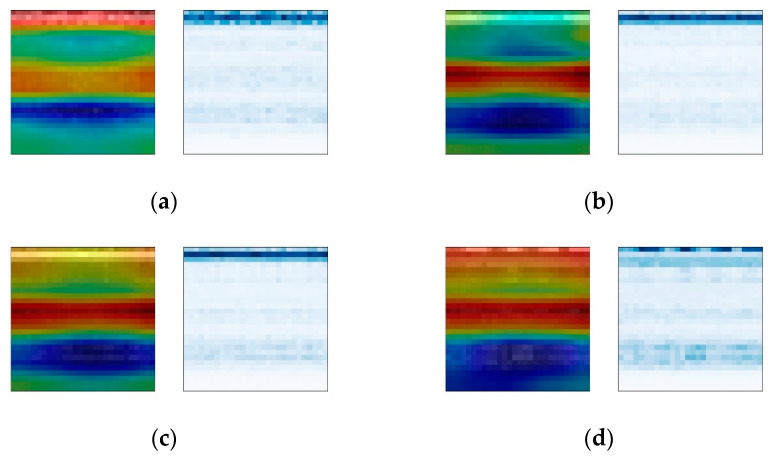
Heat maps of rolling element faults. (**a**) Rotation frequency 10 Hz; (**b**) rotation frequency 20 Hz; (**c**) rotation frequency 30 Hz; (**d**) rotation frequency 35 Hz.

**Figure 14:**
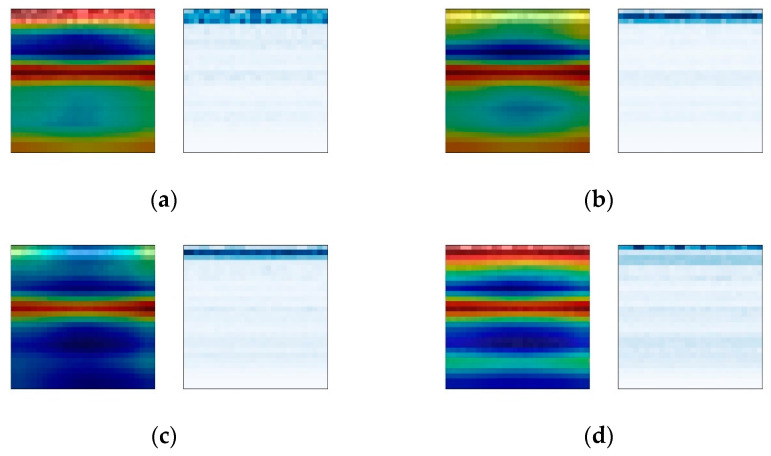
Heat maps of inner ring fault. (**a**) Rotation frequency 10 Hz; (**b**) rotation frequency 20 Hz; (**c**) rotation frequency 30 Hz; (**d**) rotation frequency 35 Hz.

**Figure 15:**
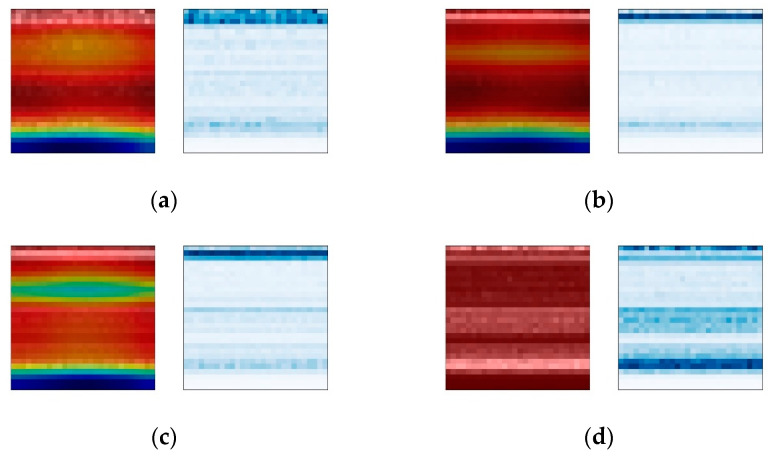
Heat maps of outer ring fault. (**a**) Rotation frequency 10 Hz; (**b**) rotation frequency 20 Hz; (**c**) rotation frequency 30 Hz; (**d**) rotation frequency 35 Hz.

The Grad-CAM module is used to analyze the visualization results of the weights of the convolutional network. For the purpose of comparison, time–frequency spectrogram samples are selected from three different fault positions at varying rotational frequencies, with a constant bearing fault severity parameter of 140 being maintained. The comparison results under different fault location conditions are shown in Figure 13, Figure 14 and Figure 15. The left images display the overlay of attention weight heatmaps with the original time–frequency spectrograms, while the right images show the corresponding original time–frequency spectrograms.

The results show that under different fault types, the CBMA-CNN model puts different levels of focus on different fault frequency bands. Due to the relatively weak amplitude of the fault signals for rolling element faults and inner race faults, the model’s attention is more distributed in the weaker frequency bands containing fault information. However, since the outer race fault is within the range of direct action of the radial load on the bearing, the impact it receives is stronger, resulting in a relatively strong overall amplitude of the vibration signal. Therefore, the model focuses on almost all frequency bands.

The comparison results under the same fault type conditions show that the model pays more attention to the frequency bands of signals in states with higher rotational frequencies. According to the calculation of the bearing fault’s characteristic frequency, the change in rotation frequency will also affect the fault signal of the bearing, and the higher the rotation frequency is, the higher the energy contained in the bearing fault signal is. Therefore, the attention changes from narrow to wide, from shallow to deep, and even extends to the new frequency bands.

In conclusion, the attention mechanism enables the fault diagnosis model to exhibit different attention focuses for different fault types and severities. The inclusion of weight visualization techniques makes the model’s attention clearer and more intuitive. Through the overlay and comparison of time–frequency plots with traditional signal analysis, a reasonable interpretation of the diagnostic mechanism of the fault diagnosis model can be achieved.

#### 4.2.3. Analysis of Model Attention Mechanism and Interpretability

**Figure 16:**
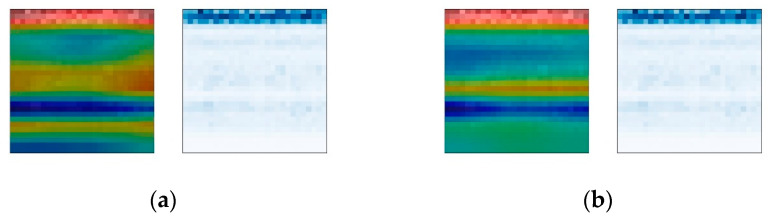
Heat maps of No. 2736 sample group. (**a**) CBAM-CNN; (**b**) Base-CNN.

To further explore the influence of the attention mechanism on the weights, a comparative experiment is carried out. In the visualization results of the Base-CNN and CBAM-CNN weights, two results of the same fault sample are selected for comparison to observe the influence of the attention mechanism on the weight changes in the fault diagnosis model. The comparison result of sample group No. 2736 is shown in Figure 16. The CBAM attention module enables the fault diagnosis model to simultaneously attend to multiple frequency bands, enhancing its capability to capture effective information in the time–frequency plot. Moreover, the attention weights of the CBAM-CNN cover the entire mid-frequency band, indicating that attention helps achieve a more uniform distribution of the model’s weights across signal frequency bands.

**Figure 17:**
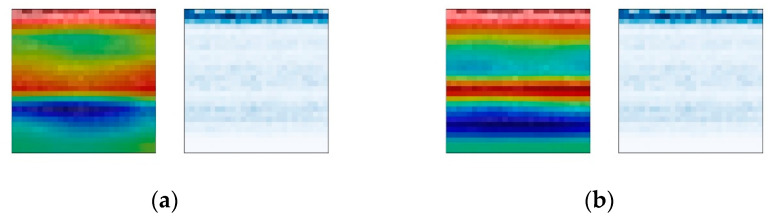
Heat maps of No. 2544 sample group. (**a**) CBAM-CNN; (**b**) Base-CNN.

The comparison result of sample group No. 2544 is shown in Figure 17. It demonstrates that the CBAM attention module enables the accurate distribution of weights in the frequency bands for the fault diagnosis model. The weights of the Base-CNN appear in the empty zone between two signal frequency bands, indicating that the Base-CNN did not accurately capture the fault information but rather overly relied on other features in the time–frequency plot for classification diagnosis. If new data are used, the diagnostic performance of the model will likely decrease.

**Figure 18:**
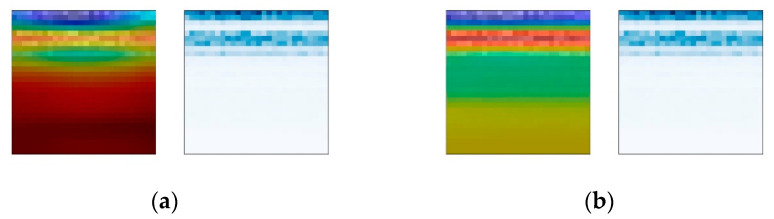
Heat maps of No. 3600 sample group. (**a**) CBAM-CNN; (**b**) Base-CNN.

The comparison results of sample group No. 3600 are shown in Figure 18. The comparison of this group shows that adding the CBAM attention module can make the weight distribution of the fault diagnosis method balanced. The weights are distributed not only in the low-frequency high-amplitude signal frequency bands but also in the high-frequency low-amplitude signal frequency bands. The weights of the Base-CNN are almost all distributed in the low-frequency and high-amplitude signal frequency bands. According to the analysis above, the middle- and high-frequency bands of the bearing contain relatively clear fault information, so the weight distribution of the CBAM-CNN is more reasonable than that of the Base-CNN. This indicates that the CBAM module can balance the distribution of weights, placing more emphasis on the frequency distribution of signals rather than the amplitude distribution. This, to some extent, mitigates the bias effect of signal amplitude on the diagnostic model.

In summary, when training with a dataset involving varying operating conditions and multiple parameter faults, the incorporation of attention mechanisms has led to a more reasonable distribution of weights in the fault diagnosis model. This aligns with conclusions drawn from traditional signal analysis, effectively improving the interpretability of the model’s fault diagnosis process.

## 5. Conclusions

In this article, a bearing fault diagnosis method based on a CBAM attention mechanism is proposed for the task of bearing fault diagnosis. This method improves the diagnostic process by introducing the Convolutional Block Attention Module (CBAM). The CBAM includes both a channel attention mechanism and a spatial attention mechanism, enabling the model to autonomously focus on learning important fault features in bearing signals during training. This addresses the issues of declining learning efficiency and diagnostic accuracy in diagnostic tasks. After completing the diagnostic task, the Grad-CAM (Gradient-Weighted Class Activation Map) weight visualization technique is used to generate heatmaps of the model’s weights. Combining this with a traditional analysis of bearing fault signals, the interpretability of the relationship between the model’s weights and bearing fault mechanisms is analyzed in the form of visual heatmaps.

The comparison results reveal that the attention module has the capability to bias the weight distribution of the fault diagnosis method towards the frequency band that is associated with the fault signal. Distinct fault types and operating conditions demonstrate varying attention focuses, exhibiting a strong correlation with the bearing fault mechanism. This offers a novel solution for interpretability research in the realm of deep learning for fault diagnosis. In summary, the proposed bearing fault diagnosis method, based on the attention mechanism, exhibits commendable diagnostic proficiency and interpretability in the task of circulatory system bearing fault diagnosis.

The proposed method has an excellent fault diagnosis ability for the signals that are affected by the bearing operating conditions and fault types. But the generalization ability of the model outside the scope of the simulated experiments has not been validated. Therefore, when the completeness of the monitoring signal dataset cannot be guaranteed, it may be worth considering improving the existing model through transfer learning. This approach aims to enable the model to quickly learn and accurately diagnose new fault signals originating from different rotating systems.

## Figures and Tables

**Table 1:** The proposed method’s structural parameters.

Number	Layer	Parameters
1	Input	size (20, 20)
2	Conv1	Input/output channel number: 1/16, kernel size: 5
3	CBAM	channel: 16, compression ratio: 8
4	Conv2	Input/output channel number: 16/16, kernel size: 5
5	FC1	size (256, 50)
6	FC2	size (50, 12)
7	Output	size (12, 1)

**Table 2:** The proposed method’s training parameters.

Number	Parameters	Content
1	Batch size	128
2	Epoch	7
3	Learning rate	Adam (lr = 0.001, betas = (0.9, 0.999))

## Data Availability

The data presented in this study are available on request from the first author or corresponding author.
